# Two-Tiered Newborn Screening with Post-Analytical Tools for Pompe Disease and Mucopolysaccharidosis Type I Results in Performance Improvement and Future Direction

**DOI:** 10.3390/ijns6010002

**Published:** 2020-01-14

**Authors:** Patricia L. Hall, Rossana Sanchez, Arthur F. Hagar, S. Caleb Jerris, Angela Wittenauer, William R. Wilcox

**Affiliations:** 1 Department of Human Genetics, Emory University, Atlanta, GA 30322, USA; rossana.sanchez@emory.edu (R.S.); alwitte@emory.edu (A.W.); william.wilcox@emory.edu (W.R.W.); 2 Georgia Department of Public Health, Atlanta, GA 30303, USA; arthur.hagar@dph.ga.gov; 3 EGL Genetics, Tucker, GA 30084, USA; sj253697@pcom.edu

**Keywords:** newborn screening, post-analytical tools, Pompe disease, mucopolysaccharidosis type I, pseudodeficiency

## Abstract

We conducted a pilot newborn screening (NBS) study for Pompe disease (PD) and mucopolysaccharidosis type I (MPS I) in the multiethnic population of Georgia. We screened 59,332 infants using a two-tier strategy of flow injection tandem mass spectrometry (FIA-MSMS) enzyme assays. The first tier of testing was a 2-plex assay measuring PD and MPS I enzyme activity, followed by a second-tier test with additional enzymes to improve specificity. Interpretation of results was performed using post-analytical tools created using Collaborative Laboratory Integrated Reports (CLIR). We identified a single case of infantile onset PD, two cases of late onset PD, and one pseudodeficiency. The positive predictive value (PPV) for PD screening during the study was 66.7%. No cases of MPS I were identified during the study period, but there were 2 confirmed cases of pseudodeficiency and 6 cases lost to follow up. The two-tier screening strategy was successful in reducing false positive results and allowed for the identification and early treatment of a case of infantile PD but the frequency of pseudodeficiency in MPS I is problematic. Molecular testing is required and should be covered by the screening program to avoid delays in case resolution.

## 1. Introduction

Pompe disease (PD, OMIM #232300) and Mucopolysaccharidosis Type I (MPS I, OMIM #607014) are the first two lysosomal storage disorders to be added to the Recommended Uniform Screening Panel (RUSP) for newborn screening (NBS) in the United States [1]. Pompe disease is a progressive, autosomal recessive lysosomal storage disorder primarily affecting skeletal and cardiac muscle. It presents with varying degrees of severity, ranging from an infantile form with cardiomyopathy and weakness before 12 months of life to later onset forms which present primarily with proximal muscle weakness, without cardiomyopathy [2,3]. MPS I is also a progressive, autosomal recessive lysosomal storage disorder with multiorgan involvement. There is great clinical variability in MPS I, with severe infantile forms that can result in death during the first decade of life and attenuated forms with significant morbidity but a near normal life span [4]. Enzyme replacement therapy is available for both disorders, and stem cell transplants are utilized to care for patients with severe MPS I. Improved outcomes have been reported with early intervention for both disorders [5].

NBS for both disorders is fully underway in many states, however it is not yet universal across the United States. These disorders are identified by measuring the activity of acid alpha-glucosidase (GAA, deficient in PD) and alpha-iduronidase (IDUA, deficient in MPS I) from dried blood spots (DBS) [5,6,7,8]. From a laboratory perspective, screening for PD and MPS I require the introduction of additional technology into the NBS lab, either MS/MS based enzyme assays [3,9,10] or digital microfluidics [6]. Primary screening using enzyme assays has been hampered by clinically benign pseudodeficiencies, particularly for MPS I [11,12]. Post-analytical tools, using the Collaborative Laboratory Integrated Reports (CLIR; https://clir.mayo.edu/) and its predecessor, Region 4 Stork (R4S) have been utilized with MS/MS screening for lysosomal storage disorders [1] and other inherited metabolic disorders [13] to improve laboratory performance over what had been previously reported. Second tier tests with high specificity, such as the quantification of glycosaminoglycans for MPSI and creatine/creatinine ratios for PD can also improve performance [1,14]. As screening panels expand across the country, care must be taken to balance the identification of true positive cases with the burden of false positive (FP) cases, both due to the impact on families who are notified of screening results, and to the health care system which must deal with these cases quickly and thoroughly.

The National Institutes of Health (NIH) have developed a program to fund states to perform pilot studies and provide information that may allow for more efficient implementation around the country. New disorders, and new technologies can be difficult to implement. Information about successful strategies can be invaluable when decisions are being made.

In the United States, Missouri has the longest history of screening for a panel of lysosomal storage disorders, having screened for a panel of five LSDs by digital microfluidics, including Pompe and MPS I, since 2013 [6]. Published data of approximately 308,000 screened infants, showed 32 cases classified as true positive for PD and 9 genotypes of uncertain significance. For MPS I, there were two confirmed cases and 2 genotypes of uncertain significance [6,15]. During the screening, there were 161 positive screens for Pompe disease and 133 for MPS I [15]. Results have also been recently published for early screening performed on infants born in Illinois [7], Kentucky [1], North Carolina [8], and New York [5]. Summary data for the performance of each of these states is shown in Table 1.

## 2. Materials and Methods

As part of the task orders for funding, the request was to screen 60,000 infants for each disorder. Initially, the decision was made to screen using digital microfluidics based enzyme assays. There were several delays with this system obtaining the appropriate regulatory approvals, as it was classified as Investigational Use Only and awaiting clearance from the Food and Drug Administration. Proceeding with this assay would have required consent under federal rules in place at the time and this was not feasible for our project and budget. Screening commenced using a laboratory developed test (LDT) with tandem mass spectrometry (MS/MS) as the detection system. This assay has been well described elsewhere [1,9,10]. We chose a custom two tier screening strategy not previously utilized by other states, and obtained custom substrate and internal standard mixes for this scheme (Perkin Elmer). The decision to proceed with the customized two-tier screening strategy was based on optimizing the screening costs and minimizing FP screens. Each enzyme screened in the reagent cocktail adds a fixed, incremental amount to the cost of the assay due to the substrate/internal standard combination (approximately $1/enzyme). The remainder of the reagents used in the sample preparation are inexpensive, and do not change with the increase in disorders. Using six enzymes in the initial step would have resulted in a 3-fold increase in fixed costs (3X more enzymes in 60,000 samples = ~$240,000 increase in reagent costs) and raised ethical questions about testing for enzymatic deficiencies but not reporting them, which we wanted to avoid. Including the 6-plex assay as a second-tier test provided a cost-effective testing strategy to reduce FP screens, while minimizing the chances of an off-target finding for one of the other enzyme analyzed.

**Figure 1:**
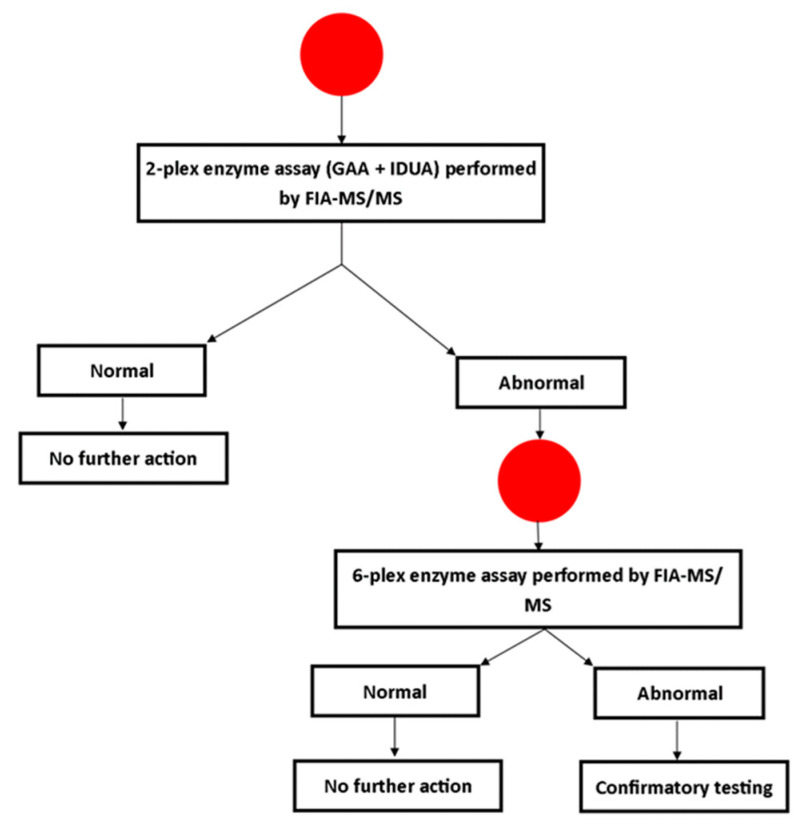
Laboratory testing algorithm for Georgia pilot study screening for MPS I and PD. FIA-MSMS: flow injection tandem mass spectrometry, IDUS: alpha-iduronidase, GAA: alpha-glucosidase.

The initial step of screening utilized a two-plex assay, measuring only GAA and IDUA activities. Any screen positives were re-analyzed using an expanded panel of six enzymes (additional enzymes included: Alpha-galactosidase (Fabry disease), acid sphingomyelinase (Niemann-Pick A/B), beta-glucosidase (Gaucher disease) and galactocerebrosidase (Krabbe disease)). The second-tier test was done on the same DBS sample. The screening algorithm developed for this program is shown in Figure 1. Data about more advanced second tier tests (dermatan and heparan sulfate in blood spots for MPS I [1], and creatine ratios for PD [14]) had not been published at the time this study was designed (2016).

Specimens were punched (⅛” punch) at the state public health laboratory and transported to the testing lab (EGL Genetics, Tucker, GA, USA; CAP/CLIA certified). Decisions about specimen quality were made by state NBS staff, using the same criteria for all other disorders screened for in Georgia. All acceptable specimens with sufficient sample remaining during the study period (January 2017–June 2017) were included in this study.

**Figure 2:**
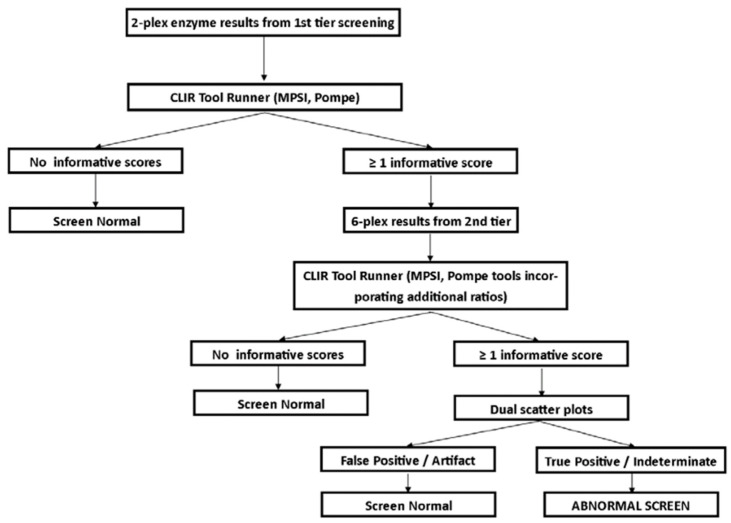
Interpretation algorithm using post-analytical tools for Georgia pilot study for MPS I and PD.

Post-analytical tools, using the Collaborative Laboratory Integrated Reports platform (CLIR; https://clir.mayo.edu/) were developed for each tier of testing. Site specific tools were created for Georgia’s screening panel, including single condition tools (PD and MPS I) for the 2-plex and 6-plex assays, and a dual scatter plot for each condition utilizing the 6-plex assay. The post-analytical tool for the first-tier test utilized the enzyme activities for GAA and IDUA, as well as the ratio between the two. The post-analytical tools for the second-tier text (6-plex) utilized the targeted enzyme for the condition, and the ratios of the other five enzymes to the targeted enzyme). Dual scatter plots utilized the same group of analytes and ratios, and the population of FP screens in the database. The interpretation algorithm used for this study is shown in Figure 2. Dual scatter plots were not used with the first tier of testing during this study to send as many samples as possible for the second-tier test, to evaluate the performance as broadly as possible. Both tiers of testing were completed from the original newborn screening sample. This strategy was designed to minimize FP screens, and reduce undue burden on the NBS system and to avoid unnecessary stress on families.

After review with the Institutional Review Boards of Emory University and the Georgia Department of Public Health, this study was deemed not to be research, as the conditions were already recommended for inclusion in the Uniform Newborn Screening Panel at the federal level and this was a study to evaluate their implementation in Georgia. The review boards determined that informed consent was not required, and the testing was conducted on all specimens submitted for routine NBS testing. Georgia’s Newborn Screening Advisory Committee also reviewed the pilot study proposal and approved.

## 3. Results

### 3.1. Screening Protocol

We screened 59,332 samples for both PD and MPS I (simultaneously). An additional punch was required for the test, however we did not encounter any samples without sufficient blood to complete the testing. Any specimens deemed to be unsatisfactory by the public health lab were designated as requiring an additional specimen before they were shipped to the testing laboratory. After the first-tier testing was completed, requests for the specimens requiring second tier testing were sent to the state public health lab. These were punched into separate plates and analyzed using the second tier, 6-plex enzyme assay. Any sample without valid results on the first-tier assay (sample loss during transport, low internal standard, ion suppression) was referred to second-tier testing. This allowed for prompt resolution of the case. Interpretation of test results for both tiers was completed utilizing CLIR post-analytical tools, as shown in Figure 2.

First tier screening results are shown in Table 1. Briefly, 285 samples had results suggestive of an analytical issue (absent results, low internal standard, sample loss or mix-up during transit) and were sent for second tier testing to allow for quick resolution. These samples were labeled as “analytical FP” results, as the results were unrelated to specimen quality or underlying physiology. 310 samples had low GAA levels and 17 had low IDUA levels. After second tier testing, all 285 analytical FP were resolved as normal, 6 screens were reported as being suggestive of PD and 11 were reported out as being suggestive of MPS I. The marked difference in screens with low GAA activity on first tier and second tier was due to many screens with minimally elevated scores using the post-analytical tools for the 2-plex. This likely could have been eliminated by using a dual scatter plot for the first tier of testing, and only sending the abnormal cases by dual scatter plot through for second tier testing. The additional granularity of the 6-plex enzyme panel was able to resolve most of these cases as normal. For MPS I, the screens that were positive on the 2-plex first tier assay had relatively greater decreases in IDUA activity compared to GAA. During the pilot study period, results for MPS I and PD did not appear on the reports generated by the NBS laboratory. This caused some confusion when reporting results to providers and families, as in many cases, they had already received a “normal NBS report” for the infant.

### 3.2. Follow-Up Testing

Confirmatory testing was recommended for screens that remained positive after the second-tier test. For PD, this was acid alpha-glucosidase measurement with reflex to urine glucose tetrasaccharide and full sequencing of *GAA* if enzyme analysis was abnormal. Infants who screened positive for PD were also recommended to have creatine kinase measurement, chest X-ray and echocardiogram (ECG) ordered. For MPS I, we recommended leukocyte alpha-iduronidase activity and urine glycosaminoglycans (quantitative and qualitative). Molecular testing of *IDUA* was deferred until the results of these initial studies had been obtained. Testing was initiated by the child’s primary care provider, and referrals to specialists were made as appropriate based on these initial test results. This follows the model for all other metabolic conditions screened for in Georgia.

### 3.3. Screening Results

Summary data for the six screens positive for PD are shown in Table 2. We identified a single case of infantile PD (P4), who had two abnormal screens. One screen was collected at approximately two hours of age, and the second screen was collected after 24 h (per local protocol, unrelated to PD result on first screen). Two cases were confirmed late onset PD (P1 and P2). One was resolved as a pseudodeficiency (P5), and one case was unresolved (P3) due to the family’s relocation out of the country. For the purposes of screening performance, these two cases are classified as FP results. The positive predictive value (PPV) of NBS for PD in Georgia was 66.7%. There have been no reported cases of false negative (FN) screening results for PD. There is a single center for medical genetics in Georgia, and it is expected that most affected infants born in the state would be referred to the medical genetics clinic at Emory University, or present to the local Children’s Hospital for care. During the study period, confirmatory testing for PD (enzyme analysis, urine biomarker, and molecular testing) was covered as part of the pilot study. Sequence analysis of *GAA* is important for proper classification and treatment of PD [16].

MPS I screen positive data are shown in Table 3. We did not identify any confirmed true positive cases of MPS I during this study. Of the 11 screens reported as positive, three infants from two families were lost to follow-up due to the family’s refusal. For MPS I follow-up testing, we followed the model we have used traditionally in Georgia, and coverage for testing was pursued through the patient’s existing insurance. This model has been effective with most conditions currently screened for in Georgia. With MPS I, we discovered a higher reliance on molecular testing to conclusively resolve cases, particularly in the context of decreased enzyme activity and normal urine glycosaminoglycan screening. While all infants who required molecular testing were able to have it performed, the delays in obtaining insurance approval were significant, and likely contributed to the reluctance of families to continue following up with genetics appointments. All infants were asymptomatic at the time of initial evaluation, which is expected even in severe cases. The family of two infants (Twins—M4 and M5) refused follow-up immediately following the screen results. As this was a pilot study to evaluate the implementation of screening, parental refusals were not escalated through state authorities as is done in some disorders included in routine NBS. After confirmatory testing, three infants (Cases M2, M6, and M7) were found to be unaffected, and two (Cases M10 and M11) were confirmed to have IDUA pseudodeficiency by sequence analysis. Pseudodeficiency alleles are more prevalent in the African American population, which makes up approximately 40% of Georgia births. Each infant with a positive screen for MPS I had at least one African American parent, making pseudodeficiency alleles a high probability contributor to our FP results. One family (Case M9) refused follow-up after undergoing confirmatory enzyme analysis, which was abnormal. Three cases (M1, M3, and M8) are classified as unresolved by genetics, however the families have missed all follow-up appointments. Two of these patients had a single uncertain variant identified each, with negative copy number analysis and the third had negative sequencing and copy number analysis. Combined with abnormal enzyme results, these findings suggest the molecular scope of pseudodeficiency and potentially disease-causing variants is not yet fully understood. There were no cases of infantile onset MPS I reported during the period covered by the pilot study. Repeat biochemical testing may be able to resolve these cases, however that has not been possible due to the families not returning to the clinic.

### 3.4. Clinical Outcomes

A single patient (P4) with infantile PD was identified during the study period and started on enzyme replacement therapy at 13 weeks. Treatment was started later than is optimal due the availability of infusion appointments. Based on *GAA* sequencing results, he was predicted to be cross-reactive immunologic material (CRIM) positive (one variant CRIM positive, one variant with unknown CRIM status) [17]. During treatment, he experienced mild infusion related reactions and underwent a local desensitization protocol. At his last visit (26 months old), his growth and development were normal for age and his most recent ECG showed normal structure and function. The two patients with late onset PD were most recently evaluated at 16 months (P1) and 24 months (P2) respectively and had no signs of disease with normal growth and development. P1 has two variants—one pathogenic and one classified as uncertain by the testing laboratory in 2016. The uncertain variant, c.868A>G (p.Asn290Asp), was described as non-pathogenic in the Erasmus database [18]. The ClinVar classification for the variant is uncertain [19]. Due to this discrepancy, the child is still being followed by genetics.

All infants with positive screens for MPS I were healthy and developing normally at their most recent evaluations. Although several have not been officially dismissed from genetics follow-up, it is believed that none of them have an early onset form of MPS I. Based on findings from other states, it is likely that these individuals are all unaffected, however the possibility of an attenuated form has not been completely ruled out.

## 4. Discussion

There are several screening strategies that have been proposed for use with the enzyme assays used for LSDs in NBS. Post-analytical tools, fixed cutoffs and cutoffs based on the daily mean have all been used. Based on previously published data, and the results of our study (Table 1), any evidence based, appropriately validated screening strategy should detect all true positive cases reliably, with variations in the number of FP results introduced into the screening system. For MPS I, the most effective second tier test is likely quantitation of dermatan and heparan sulfate in blood spots, as used in the screening of infants born in Kentucky [1]. Due to the high prevalence of pseudodeficiency alleles, and the reduction in enzyme activity associated with them, additional enzymes as a second tier test was not sufficient to reduce FP screens. North Carolina’s MPS I post hoc analysis of their screening showed similar performance to Georgia, showing that sequence analysis of *IDUA* as a second tier test did not reduce FP results beyond additional enzymes being analyzed [8]. For PD, the PPV was significantly better than states who screened using some variation of cutoffs, whether it was a fixed cutoff or the percentage of the daily mean [7,15]. The PPV for PD compared favorably to the results of screening Kentucky newborns performed by Mayo Clinic using similar post-analytical tools [1,14].

As new conditions are added to screening panels aggressive management of laboratory performance particularly with respect to PPV, needs to be considered. Our 2-plex approach for the first-tier test offers cost-savings compared to the first tier 6-plex approach utilized for the Kentucky screening, as the reagent cost of the 2-plex is approximately ⅓ of the 6-plex. Other variations of the second-tier test (with 3, 4 or 5 enzymes) are possible, however there is unlikely to be a significant decrease in total costs, if this strategy refers more children for follow-up. The power of post-analytical tools is greatest when multiple sites can collaborate and share data to increase the population of cases for rare disease.

The pilot study provided valuable data for the decision makers involved with Georgia’s NBS program. In May 2018, the Commissioner of Public Health approved the recommendation for these conditions to be added to the state’s NBS screening panel, contingent upon proper funding being provided for the screening and follow-up process, including molecular testing where appropriate. This funding was approved in the budget for fiscal year 2020 (July 2019–June 2020). The difficulty in getting insurance approvals for timely molecular analysis of *IDUA* was one of the major roadblocks encountered in this study, and the inclusion of funding from the NBS program should resolve this and ensure appropriate follow-up for all infants identified by NBS. Since the conclusion of the pilot study, at least two patients with infantile PD and two patients with early onset MPS I were born in Georgia. All of these cases came to the attention of the medical genetics clinic at Emory University and all had a significant gap between birth and diagnosis. The clinical identification of these patients during the post-pilot period has increased confidence that there were no FN for infantile onset disease during the pilot study. Missed cases of later onset forms may not be ascertained for years.

A two-tiered screening strategy offers several advantages in the NBS setting. The use of a lower specificity test on the first tier allows for aggressive filtering based on these results to identify possible true positive cases, which can be refined by proper use of the second tier test to only report out those cases with the highest probability of being a true positive. This strategy has proven effective for cystic fibrosis, congenital adrenal hyperplasia, maple syrup urine disease, and remethylation disorders [20]. Given the potential burden to the health system of screen positives for LSDs, reduction of FP screens should be a priority. The screening strategy we utilized for our pilot study combines a lower specificity first tier test with a more expensive, and higher specificity second tier test, and post-analytical tools to take advantage of multiple analytes included in screening. The most effective screening strategy for PD and MPS I differed in this study. Our strategy utilizing an expanded panel of enzymes with post-analytical tools provided good performance for PD, but resulted in a high number of FP results for MPS I. Based on this study, and other published NBS results, the most effective second tier test for MPS I is likely LC-MS/MS analysis of dermatan and heparan sulfate [1]. These strategies can result in savings in the NBS lab setting, which is significant due to the fact that many NBS labs do not have direct control over their fees. While the pilot studies were successful in Georgia, full screening has not yet been implemented. One of the barriers to implementation was the difficulty in obtaining insurance coverage for molecular testing required to resolve all screen positive cases in an appropriate timeline. We also experienced issues with follow-up by families when presented with uncertain results and possible late onset conditions. This information was valuable for the state NBSAC in making its recommendations to add conditions and provide funding for testing to the Commissioner of Public Health.

## Figures and Tables

**Table 1:** Published results of US-based newborn screening (NBS) programs screening for Pompe disease (PD) and mucopolysaccharidosis type I (MPS I). Georgia data from this study are included in the last row.

Region Screened	Methodology	Interpretation	Disorder	# Screened	Screen Positive	True Positives	PPV (%)	Screen Positive Rate (%)
Illinois [7]	MSMS (1 tier)	% daily median	Pompe	219,973	139	10	7.2	0.06
			MPS I	219,973	151	1	0.7	0.07
Kentucky [1]	MSMS (2 tier)	Post-analytical tools	Pompe	55,161	2	2	100.0	<0.002
			MPS I	55,161	2	1	50.0	<0.002
Missouri [6]	Digital microfluidics	Cutoff	Pompe	~308,000	161	32	19.9	0.05
			MPS I	~308,000	133	2	1.5	0.04
New York [5]	MSMS (1 tier)	% daily mean	Pompe	18,105	6	1	16.7	0.03
			MPS I	35,816	13	0	0.0	0.04
North Carolina [8]	MSMS (2 tier) + Sequencing	Cutoff (Initial)	MPS I	62,734	54	1	1.9	0.09
Georgia	MSMS (2 tier)	Post-analytical tools	Pompe	59,332	6	4	66.7	0.01
			MPS I	59,332	11	0	0.0	0.02

**Table 2:** Positive screens for PD during Georgia pilot study. Collaborative Laboratory Integrated Reports (CLIR) interpretation possibilities are uninformative (below 1st percentile), possible (1st–5th percentiles), likely (5th–20th percentiles) and very likely (>20th percentile). * Enzyme units are pmol/punch/hour. Enzyme interpretations are those reported by the laboratory performing confirmatory analysis. All GAA confirmation results were performed on DBS. Reference range is >3.88 pmol/punch/hour. Variant interpretations are those received from the clinical laboratory performing testing (B = benign; P = pathogenic; U = variant of uncertain significance). ** Classified as non-pathogenic in Erasmus database in 2012. See discussion in text.

Case ID	Birth Weight (g)	Sex	Race	Age at Collection	DBS GAA	CLIR Score (Interpretation)	GAA Confirmation *	Glucose Tetrasaccharde	Allele 1	Allele 2	Outcome
P1	3695	F	Black	24	2.555	37 (Possible)	2.2 Abnormal	Normal	c.868A>G p.Asn290Asp (U) **	c.2105G>A p.Arg702His (P)	Late onset PD
P2	3515	F	White	24	1.677	127 (Likely)	3.9 Abnormal (per lab)	Normal	c.-32-13T>G (P)	c.2238G>C p.Trp746Cys (P)	Late onset PD
P3	2570	M	Black	114	1.026	103 (Possible)	Family refused follow-up				
P4	3360	M	Black	2	1.581	32 (Possible)	2.7 Abnormal	Elevated	c.1755-1G>A (P)	c.2236T>C p.Trp746Arg (P)	Infantile onset PD
				56	1.103	127 (Likely)					
P5	2560	M	Native Hawaiian/Pacific Islander	33	1.019	127 (Likely)	2.7 Abnormal	Normal	c.1726G>A/c.2065G>A p.Gly576Ser (B)/p.Glu689L (B)	c.1726G>A/c.2065G>A p.Gly576Ser/p.Glu689L	Pseudodeficiency

**Table 3:** Positive screens for MPS I during Georgia pilot study. CLIR interpretation possibilities are uninformative (below 1st percentile), possible (1st–5th percentiles), likely (5th–20th percentiles) and very likely (>20th percentile). Variant interpretations are those received from the clinical laboratory performing testing (B = benign; U = variant of uncertain significance). * Enzyme results interpreted by testing laboratory as abnormal; laboratory specific reference ranges are shown in parentheses. ** Cases 4 and 5 are twin siblings.

Case ID	Birth Weight (g)	Sex	Race	Age at Collection (hours)	DBS IDUA	CLIR Score (Interpretation)	IDUA Confirmation	Glycosaminoglycans	Allele 1	Allele 2	CNV Analysis	Outcome
M1	3412	M	Black	32	0.194	346 (Likely)	0.6 nmol/h/mL (≥1.0) *	Normal	c.923T>C p.Leu308Pro (U)	None	Negative	Unresolved (Lost to Genetics Follow-up)
M2	2994	F	Black	24	0.355	177 (Likely)	4.2 nmol/h/mg protein (3.57–21.40)	Essentially Normal (Repeat Normal)	Not performed			Unaffected
M3	3470	M	Black	24	0.238	337 (Likely)	7.4 pmol/punch/h (>8.0)	Normal	C.1238_1264del (U)	None	Negative	Unresolved (Lost to Genetics Follow-up)
M4 **	2320	M	Black	49	0.186	266 (Likely)	Family refused follow-up					
M5 **	2360	M	Black	50	0.226	228 (Likely)	Family refused follow-up					
M6	3820	F	Black	25	0.597	112 (Likely)	5.7 nmol/h/mg protein (3.57–21.40)	Normal	Not performed			Unaffected
M7	3030	M	Black	40	0.222	335 (Likely)	5.1 nmol/h/mg protein (3.57–21.40)	Normal	Not performed			Unaffected
M8	2817	F	Black	25	0.302	138 (Likely)	1.6 nmol/h/mg protein (12.0–65) *	Normal	None	None	Negative	Unresolved (Lost to Genetics Follow-up)
M9	3165	F	White	25	0.242	266 (Likely)	0.3 nmol/h/mg protein (3.57–21.40) *	Family refused further follow-up				
M10	2906	F	Black	25	0.403	212 (Likely)	0.3 nmol/h/mL (≥1.0) *	Normal	c.235G>A p.Ala79Thr (B)	c.235G>A p.Ala79Thr (B)	Not performed	Pseudodeficiency
M11	3375	M	Black	50	0.323	200 (Likely)	0.2 nmol/h/mg protein (3.57–21.40) *	Normal	c.235G>A p.Ala79Thr (B)	c.235G>A p.Ala79Thr (B)	Not performed	Pseudodeficiency
